# Is Obesity in Young People With Psychosis a Foregone Conclusion? Markedly Excessive Energy Intake Is Evident Soon After Antipsychotic Initiation

**DOI:** 10.3389/fpsyt.2018.00725

**Published:** 2018-12-24

**Authors:** Scott B. Teasdale, Philip B. Ward, Rebecca Jarman, Tammy Wade, Elisa Rossimel, Jackie Curtis, Julia Lappin, Andrew Watkins, Katherine Samaras

**Affiliations:** ^1^Keeping the Body in Mind Program, South Eastern Sydney Local Health District, Sydney, NSW, Australia; ^2^School of Psychiatry, UNSW Sydney, Sydney, NSW, Australia; ^3^Schizophrenia Research Unit, South Western Sydney Local Health District, Ingham Institute for Applied Medical Research, Liverpool, NSW, Australia; ^4^Faculty of Health, University of Technology Sydney, Ultimo, NSW, Australia; ^5^Department of Endocrinology, St Vincent's Hospital, Sydney, NSW, Australia; ^6^Diabetes and Metabolism Division, Garvan Institute of Medical Research, Sydney, NSW, Australia; ^7^St Vincent's Clinical School, UNSW Sydney, Sydney, NSW, Australia

**Keywords:** weight gain, psychosis, antipsychotics, early intervention, diet

## Abstract

**Introduction:** Antipsychotic medication (APM) initiation is associated with rapid and substantial weight-gain and high rates of obesity. Obesity leads to premature onset of cardiometabolic diseases and contributes to the 15–20 year shortfall in life expectancy in those experiencing severe mental illness. Dietary energy intake excess is critical to weight management but is yet to be quantified in youth with first episode psychosis (FEP) receiving APM. This study aimed to describe the degree of energy overconsumption and the food sources contributing to this in youth with FEP.

**Materials and Methods:** People aged 15–30 years with FEP receiving APM completed diet histories through qualified dietitians to assess energy imbalance and food sources. Outcome measures were: (i) energy balance; and (ii) intake of core and discretionary foods.

**Results:** Participants (*n* = 93) were aged 15–29 years (mean = 21.4 ± 2.9 years) and exposed to APMs for a median for 8 months (Interquartile Range (IQR) 11 months). Energy balance was exceeded by 26%, by a median 1,837 kJ per day (IQR 5,365 kJ). APM polypharmacy and olanzapine were linked to larger excesses in dietary energy intake. The greatest contributors to energy intake were refined grain foods (33%) and discretionary foods (31%).

**Conclusion:** Young people with FEP receiving APMs appear to have markedly excessive energy consumption, likely contributing to rapid weight-gain, and thereby seeding future poor physical health. Larger, prospective studies are needed to gain a greater understanding of dietary intake, and its effects on health, in people with FEP.

## Introduction

People receiving antipsychotic medication (APM) for first-episode of psychosis (FEP) experience rapid, excessive weight gain and acquire risk factors for cardiometabolic disease (1, 2). Weight-gain is most rapid in the first few months of APM treatment, and accompanied by central obesity (3). Longitudinal data shows that mean weight gain is 12 kg over the first 2 years of APM treatment, increasing to a mean 19 kg over the first 4 years (4). The higher rates of abdominal obesity (OR 4.43), hypertriglyceridemia (OR 2.73), metabolic syndrome (OR 2.35), low HDL (OR 2.35), diabetes (OR 1.99), and hypertension (OR 1.36) compared to controls (5), culminates in a 20-year life expectancy gap compared to the general population (6, 7). Each of these drivers ill health are preventable lifestyle factors relating to dietary intake.

A recent prospective study followed people from their first episode of psychosis, and found that after 20 years, 62% of people with schizophrenia and 50% of people with bipolar disorder were obese, substantially higher than national averages (8). Interestingly, those with SCZ gained significantly more weight in the first 10 years compared to the subsequent 10 years, whereas those with bipolar disorder gained less weight in the first 10 years compared to years 10–20. Critically, this study monitored weight change for 20 years, significantly longer than other prospective studies, and showed for the first time that weight gain may not being to plateau until between 10 and 20 years post first hospitalization. The authors also suggested that participants could continue to gain weight, even after the 20-year observation period.

Appropriate dietary energy intake is fundamental to weight stability, management of weight excess, and managing cardiometabolic risk. It is yet to be explored, however, in people with FEP. APM are associated with increased appetite (9), unhealthy dietary intake (10), disordered eating behaviors (11) and sedentary behavior (reduced energy expenditure) (12), however mechanisms are not clearly understood. Dopamine, serotonin, muscarinic, and histamine receptors have all been implicated in APM-induced hunger changes, with APM with a high affinity for the 5-HT_2C_ and muscarinic receptors associated with the greatest risk for weight-gain (13). Evidence also suggests weight-gain mechanisms innate to the psychiatric illness, such as brain structural damages (14). The eating process in those experiencing psychosis is made difficult due to impaired executive functioning, which complicates restrained eating and facilitates disinhibition (15). It appears that both drug-naïve and those receiving APM treatment have insensitive rewards systems shifting the preference to less nutritious foods high in sugar, salt, and fat (14).

Whilst APM-induced changes in appetite, weight and obesity have been well-documented, the energy intakes, food preferences and dietary quality of youth with FEP receiving APM have yet to be clearly quantitated. Limited published data highlight unhealthy dietary habits in severe mental illness (10), with diets lower in fruit and fiber, and higher intake of sweet foods and drinks compared to the general population. Much remains to be delineated, including quantitation of energy excess, dietary quality, and shortfalls to national dietary guidelines.

In addition to energy consumption, diet quality also importantly contributes to health. Quality is inferred from the inclusion of essential fatty acids, protein, vitamins, minerals, and fiber, typically found in “core food groups” such as vegetables, fruit, wholegrains, milk, cheese, and yogurt, and protein-rich foods (meat, poultry, seafood, eggs, nuts, and legumes). On the other hand, “discretionary foods” are low- or non- nutritious foods typically found in “Westernized” diets, which are generally high in added sugars, salt and/or fat, and are identified risk factors for poor cardiometabolic health (16).

Understanding these food choices and preferences is fundamental to the development of dietary strategies to reverse and/or prevent APM-induced obesity in youth with psychosis. To the authors' knowledge no study has yet examined energy intake relative to individual energy requirement in order to estimate energy excess, in the early stage of APM-treatment when weight gain occurs most rapidly. Further, comparisons of diet quality against national standards are lacking.

## Aims of the Study

In youth with FEP receiving APM, this study aimed to (i) quantify energy intake against energy requirements to determine the degree of energy intake imbalance, and (ii) examine diet quality, compared to Australian national dietary recommendations.

## Methods

### Design

We undertook a cross-sectional analysis of dietary intake in youth experiencing FEP. Inclusion criteria were: (i) 15–30 years of age, (ii) within 2 years of first onset of psychotic symptoms, and (iii) receiving APM. Exclusion criteria were having received dietary education and/or intervention since commencing APM. The treating psychiatrists provided data on psychotropic medications and Diagnostic and Statistical Manual for Mental Disorders (DSM-V) diagnoses (17). This study received ethical approval from the South Eastern Sydney Local Health District Human Research Ethics Committee (HREC ref no: 14/276; LNR/14/POWH/614). This study was reported using the Strengthening the Reporting of Observational Studies in Epidemiology–Nutritional Epidemiology (STROBE-nut) guidelines (18).

### Participants/Setting

Participants were patients who were referred, as part of routine care, to the *Keeping the Body in Mind* program between January 2015 and December 2017, from three allied community youth mental health services in urban Sydney. *Keeping the Body in Mind* is a lifestyle program involving a dietitian, exercise physiologist, and mental health nurse consultant, targeting the physical health of people with mental illness. On referral to the program, dietary intake was assessed by a dietitian using a comprehensive diet history (19), accounting for usual intake and variation. A food checklist was included to ensure all food categories were evaluated. Portion size was estimated using food models and measuring cups. The frequency of various foods was evaluated, including the typical composition of meals (20). Individual diet histories were completed in a private consulting room, over 30–60 min. This dietary assessment formed part of a broader cardiometabolic lifestyle assessment (21), including anthropometric and physical activity measures and was conducted prior to the delivery of *Keeping the Body in Mind* intervention (22).

### Outcome Measures

Estimated energy intake (EEI) was derived from kilojoule values assigned to food groups described in the Australian Guide to Healthy Eating (AGHE) (23). To determine energy balance, estimated energy requirement (EER) was calculated for each participant using the Schofield equation (24), adjusting for physical activity level (sedentary 1.3–1.4x BMR, light-moderate 1.5–1.6x BMR, active 1.7–1.8x BMR) (25) and using adjusted ideal body weight for participants with a body mass index (BMI) ≥25 kg/m^2^ (see Supplementary Material 1) (26). Using Goldberg et al. cut-off limit for plausible intake, participants were considered to be underreporting if the EEI:BMR ratio was <0.9 (27).

Diet quality was measured by estimating serves of core food groups: (i) vegetables (separately for starch and non-starch vegetables), (ii) fruit, (iii) milk, cheese, yogurt, and alternatives, (iv) grain foods, (v) protein foods such as meat, poultry, fish, eggs, nuts and seeds, and (vi) discretionary foods as defined in the AGHE (23). These estimated intakes of food group servings were compared to the recommended serves, based on age and sex, also defined in the AGHE (23). Grain foods were further categorized into mostly wholegrain (≥50% wholegrain products) or refined (<50% wholegrain products).

Weight (kg) was measured with participants barefoot in light street clothing. Height (m) was measured using a stadiometer with participants barefoot. Body mass index (BMI) was calculated using weight/ height x height, kg/m^2^. Waist circumference (cm) was measured horizontally at the navel using a measuring tape to the nearest 0.1 cm. BMI was categorized using the World Health Organization classification (28). Central obesity was categorized using waist circumference and the International Diabetes Federation classification (29).

### Statistical Analyses

We compared (i) estimated energy intake and recommended energy intakes, and (ii) estimated food group intake and recommended food group intakes. The Shapiro-Wilk test was run as a test of normality. Paired sample and/or independent samples *t*-tests were run to test for significance between variables for normally distributed data, and reported as mean and standard deviation. The Wilcoxon Signed Ranks Test was used to test for statistical significance for non-normally distributed data. Non-normally distributed data were reported as median and range. Chi-squared tests examined the weight and waist circumference status of participants. Kruskal-Wallis Tests with Pairwise Comparisons were used for subgroup analyses of excess energy intake by diagnosis and APM prescription. A Spearman's Rho correlation was run between excess energy intake and: (i) chlorpromazine equivalents, and (ii) duration of APM treatment. Statistical significance was set at *p* < 0.05. A Bonferroni correction was incorporated for *t*-test comparisons across multiple food groups, with a new statistical significance level set at *p* < 0.007. Analyses were performed using SPSS Version 24 (Chicago, IL, United States).

## Results

There were 93 participants (58 males, 62%), mean age 21.4 ± 2.9 years. Participants were predominantly Europids (*n* = 52, 56%) and, in decreasing frequency, Asian (*n* = 27, 29%), Maori/Pacific Islander (*n* = 6, 7%), Aboriginal/Torres Strait Islander (*n* = 3, 3%), Middle Eastern (*n* = 3, 3%), South American (*n* = 1, 1%), and African (*n* = 1, 1%). DSM-V diagnoses were: schizophrenia (*n* = 32), bipolar affective disorder (*n* = 16), psychosis not otherwise specified (*n* = 15), major depressive disorder with psychosis (*n* = 10), schizoaffective disorder (*n* = 10), substance-induced psychosis (*n* = 4), schizophreniform disorder (*n* = 2), delusional disorder (*n* = 1), organic psychosis (*n* = 1), brief reactive psychosis (*n* = 1), and psychosis due to another medical condition (*n* = 1). Demographic, diagnostic and medication data are provided in Table 1.

**Table 1:** Demographic and clinical measures in young people with first episode psychosis.

		**Males(*n* = 58)**	**Females (*n* = 35)**	**Total(*n* = 93)**
**DEMOGRAPHIC**				
Age	mean (SD)	21.5 (2.8)	21.2 (3.2)	21.4 (2.9)
**Ethnicity**	*n* (%)			
Europid		37 (64)	15 (42)	52 (56)
Asian		13 (21)	14 (40)	27 (29)
Maori/Pacific Islander		5 (9)	1 (3)	6 (7)
Aboriginal/Torres Strait Islander		1 (2)	2 (6)	3 (3)
Middle Eastern		1 (2)	2 (6)	3 (3)
African		0 (0)	1 (3)	1 (1)
South American		1 (2)	0 (0)	1 (1)
**PHYSICAL ACTIVITY LEVEL**				
Sedentary		36 (62)	24 (68)	60 (64)
Light-Moderate		14 (24)	10 (29)	24 (26)
Active		8 (14)	1 (3)	9 (10)
**ANTHROPOMETRIC MEASURES**				
Weight	kg (SD)	82.4 (15.4)	66.8 (16.2)	76.5 (17.4)
BMI	kg/m^2^ (SD)	26.1 (4.4)	24.9 (5.7)	25.7 (5.0)
**BMI classification**	n (%)			
Underweight (<18.5 kg/m^2^)		1 (2)	0 (0)	1 (1)
Ideal weight (18.5–24.9 kg/m^2^)		22 (38)	23 (66)	45 (49)
Overweight (25–29.9 kg/m^2^)		27 (46)	4 (11)	31 (33)
Obese (≥30.0 kg/m^2^)		8 (14)	8 (23)	16 (17)
Waist circumference	cm (SD)	93.7 (12.6)	86.7 (12.8)	91.0 (13.0)
**Waist circumference classification**	n (%)			
Ideal		28 (48)	13 (37)	41 (44)
Increased risk		30 (52)	22 (63)	52 (56)
**DSM-V Diagnosis**	n (%)			
Schizophrenia		25 (43)	7 (20)	32 (34)
Schizoaffective disorder		5 (8)	5 (14)	10 (11)
Schizophreniform disorder		0 (0)	2 (6)	2 (2)
Bipolar affective disorder		9 (16)	7 (20)	16 (17)
Substance-induced psychosis		3 (5)	1 (3)	4 (5)
Psychosis not otherwise specified		6 (10)	9 (25)	15 (16)
Major depression with psychosis		9 (16)	1 (3)	10 (11)
Delusion disorder		0 (0)	1 (3)	1 (1)
Organic psychosis		1 (2)	0 (0)	1 (1)
Brief reactive psychosis		0 (0)	1 (3)	1 (1)
Psychosis due to another medical		0 (0)	1 (3)	1 (1)
Condition				
**PSYCHOTROPIC MEDICATIONS**				
**Antipsychotic**	n (%)			
Risperidone		18 (31)	10 (29)	28 (30)
Aripiprazole		12 (21)	9 (26)	19 (20)
Quetiapine		4 (7)	4 (11)	8 (9)
Olanzapine		11 (19)	5 (14)	16 (17)
Clozapine		3 (5)	0 (0)	3 (3)
Amisulpride		1 (2)	1 (3)	2 (2)
Ziprasidone		1 (2)	1 (3)	2 (2)
Paliperidone		2 (3)	2 (6)	4 (4)
Antipsychotic polypharmacy		6 (10)	5 (14)	11 (12)
Chlorpromazine equivalent	mg (SD)	261 (208)	209 (138)	242 (186)
Duration of antipsychotic (months)	median (IQR)	8.7 (7.5)	7.0 (5.7)	8.1 (6.9)
**Antidepressant**	n (%)			
Yes		15 (26)	12 (34)	27 (29)
**Mood stabilizer**				
Yes		15 (26)	5 (14)	20 (22)
**Benzodiazepine**				
Yes		4 (7)	1 (3)	5 (5)
**ADDITIONAL MEDICATION**				
Metformin		1 (2)	1 (3)	2 (2)

The majority of participants received APM monotherapy (*n* = 81, 88%): risperidone (*n* = 28), aripiprazole (*n* = 19), olanzapine (*n* = 16), quetiapine (*n* = 8), paliperidone (*n* = 4), clozapine (*n* = 3), amisulpride (*n* = 2), ziprasidone (*n* = 2). Eleven participants were prescribed dual APM: aripiprazole with olanzapine (*n* = 5), risperidone (*n* = 1), or quetiapine (*n* = 2); clozapine with amisulpride (*n* = 1), aripiprazole and quetiapine (*n* = 1); and quetiapine with paliperidone (*n* = 1). Mean dosage prescribed was 242 ± 186 mg chlorpromazine equivalents. Median APM exposure was 8 months (IQR 7 months). A range of mood stabilizer, antidepressant and benzodiazepine medications were also prescribed to some participants.

The mean BMI was 25.7 ± 5.0 kg/m^2^ (males: 26.1 ± 4.4 kg/m^2;^ females 24.9 ± 5.7 kg/m^2^). Forty three participants had healthy BMI (48%), with large proportions overweight (*n* = 31, 33%) or obese (*n* = 16, 17%). One participant was underweight. Males (60%) were more frequently overweight or obese compared to females (34%), (*X*^2^ = 5.9, *p* = 0.01). The percent of females with central obesity was numerically higher than for males (*n* = 22, 63% vs. *n* = 30, 52%), but this difference was not statistically significant, *X*^2^ = 1.1, *p* = 0.2).

All participants met Goldberg's criteria for plausible energy intake reporting. Estimated energy intake (EEI) was significantly and substantively higher than estimated energy requirements (EER) (*Z* = −5.1, *P* < 0.001), with a median energy intake excess of 1,837 kJ per day (IQR 5,365 kJ). Median energy intake excess for males was 1,771 kJ per day (IQR 5,355 kJ), and for females 1,837 kJ per day (IQR 5,196 kJ) (Table 2). There was a significant difference for mean excess energy between different APM (*H* = 15.7, *p* < 0.05). Excess energy consumption was significantly higher in those prescribed APM polypharmacy when compared to those prescribed amisulpride (*p* < 0.01) and those prescribed risperidone (*p* < 0.05); those prescribed olanzapine reported significantly higher excess energy intake when compared to amisulpride (*p* < 0.01), risperidone (*p* = 0.02) and aripiprazole (*p* = 0.04). There was no relationship between medication dosage (chlorpromazine equivalents), or duration of treatment with APM, and excess energy intake in this sample (*r* = −0.004, *p* = 0.97; *r* = 0.02, *p* = 0.85). There was no significant difference between those prescribed: one APM only, APM polypharmacy, APM and mood stabilizer, APM and antidepressant, or APM, mood stabilizer and antidepressant (*X*^2^ = 3.8, *p* = 0.44). There was no significant difference between all diagnoses within this sample (*H* = 2.3, *p* = 0.81), or major subgroups: (i) schizophrenia spectrum, (ii) bipolar affective and (iii) major depression with psychosis (*H* = 0.6, *p* = 0.90).

**Table 2:** Energy intake in young people with first episode psychosis.

	**Mean energy intake kJ/day (SD)**	**Mean energy requirement kJ/day (SD)**	**Median difference kJ/day (IQR)**	**Wilcoxon signed ranks test**
**GROUP**				
Male	13,651 (4,517)	11,167 (1,320)	1,771 (5,355)	*Z* = –3.6, *p* < 0.001
Female	11,513 (4,326)	8,479 (1,132)	1,837 (5,196)	*Z* = –3.8, *p* < 0.001
Total	12,846 (4,544)	10,155 (1,808)	1,837 (5,365)	*Z* = –5.1, *p* < 0.001

The sources of energy intake were, in descending order of magnitude: grain foods (33%); discretionary foods (31%); protein-rich foods (17%); added unsaturated fats and oils including spreads (6%); dairy (milk, cheese and yogurt) (6%); fruit (4%); and vegetables (3%). Mean daily serves of food groups were: grain foods 7.2 ± 3.7, discretionary foods 6.6 ± 5.8, protein foods 3.7 ± 2.2, dairy foods 1.7 ± 1.4, fruit 1.7 ± 1.8, and vegetables 2.8 ± 1.8 (Table 3).

**Table 3:** Description of dietary patterns in young people with first episode psychosis.

	**Percent of energy intake**	Recommended servings/day^*^		**Mean intake servings/day (SD)**		Statistical test ^**^	**Percent of participants meeting national nutrition recommendations ^(11)^**.	
		**Male**	**Female**	**Male (*n* = 22)**	**Females (*n* = 13)**	
**FOOD GROUPS**							
Grain foods	33	6	6	7.1 (3.8)	7.4 (3.8)	t_(92)_ = 2.5, *p* = 0.013	19% choosing predominantly wholegrain
Protein-based foods	17	3	2.5	4.0 (3.2)	3.2 (1.9)	t_(92)_ = 2.8, *p* = 0.009	
Dairy and alternatives	6	2.5	2.5	1.8 (1.5)	1.6 (1.2)	t_(92)_ = −6.5, *p* < 0.001^***^	27%
Vegetables	3	6	5	2.6 (1.8)	3.3 (1.8)	t_(92)_ = −13.4, *p* < 0.001^***^	14%
Fruit	4	2	2	1.8 (2.2)	1.5 (1.2)	t_(92)_ = 1.6, *p* = 0.103	39%
Oils/spreads	6	4	2	2.0 (1.3)	1.8 (1.5)	t_(92)_ = −14.9, *p* < 0.001^***^	28%
Discretionary foods	31	0–3	0–2.5	7.6 (6.6)	4.8 (3.3)	t_(92)_ = 6.4, *p* < 0.001^***^	27%

* *Recommended serves per day including serving sizes are described in the Australian Guide to Healthy Eating ^(11)^*.

** *Mean intake of all participants compared to recommended serves/day*.

*** *Statistically significant after applying Bonferroni correction, with statistical significance set at p < 0.007*.

Reported intakes were compared to Australian national dietary recommendations. There was a significantly higher than recommended intake of discretionary [*t*_(92)_ = 6.4, *p* < 0.001]. In contrast, there were shortfalls in recommended intakes of vegetables [*t*_(92)_ = −13.4, *p* < 0.001], dairy [*t*_(92)_ = −6.5, *p* < 0.001], and unsaturated oils/spreads [*t*_(34)_ = −14.9, *p* < 0.001]. The majority of participants consumed grain-containing foods as refined/processed products (81%); only 19% consumed predominantly wholegrain products. The majority of participants had shortfalls in the recommended intakes of vegetables (86%), dairy (73%), and fruit (61%). The recommended intake of discretionary food intake was exceeded in 72% of participants.

## Discussion

To our knowledge, this is the first study to demonstrate that youth with severe mental illness receiving APM and other psychotropic medications have energy intakes that exceed individual requirements by some 26% on average. Further, excessive intakes of discretionary foods and refined grain-based foods were evident, along with insufficient intakes of vegetables, dairy and fruit. In combination with the sedentariness already documented in people with psychosis (12), this excessive energy intake is likely to contribute substantially to the rapid and excessive weight-gain observed in the years after APM initiation. The weight change dynamics paradigm of Hall and co-workers' (30) predicts that every 100 kJ (24 kcal) intake excess will have an eventual bodyweight change of 1 kg, with half this weight change occurring within 1 year, and 95% weight change occurring within 3 years. Applying this model to the excess daily energy intake of 1,837 kJ (439 kcal) found in our study an eventual bodyweight change of approximately 18 kg would be expected. This can help explain the mean 20 kg weight gain that has been observed in people receiving APM over the first 4 years of treatment (4).

Whilst increasing physical activity is important for reducing cardiovascular risk, and symptoms of depression and possibly psychosis (31, 32), increased physical activity alone is unlikely to adequately address this excessive energy imbalance: the hypothetical 70 kg person would need to walk an additional 3.5 h at 4 km/h or run for 1 h at 10 km/h each day to expend the observed energy surplus (33). Therefore, reduction of energy intake will be essential for preventing or reducing the weight gain that follows APM initiation and their long-term use.

The higher excess in dietary energy intake found for both APM polypharmacy and olanzapine is congruent with the literature, with both of these having higher weight gain potential compared to other second-generation APM (34, 35). Treatment with a mood stabilizer and some antidepressant medications can result in weight-gain. Given that our inclusion criteria were young people with FEP receiving APM, with no restriction on additional psychotropic medication prescription, it was difficult to disentangle the effects of mood stabilizer and antidepressant medication on dietary intake. Larger studies, with set psychotropic medication criteria would allow greater exploration of the effects of mood stabilizer and antidepressant medications (with and without APM) on food and dietary energy intake. Our study did not find a relationship between APM dosage and excess dietary energy intake, although it is conceivable that higher APM dosage would be associated with greater appetite and therefore higher dietary energy intake. Larger, appropriately designed studies would be needed to confirm the effect that APM dosage has on dietary intake.

Study limitations include the following: First, the cross-sectional design means that prospective studies that measure dietary intake at multiple time points are needed to confirm these findings. Second, while the program aims to assess all young people with FEP attending local health district services, there is potential for more health conscious consumers to engage in a dietary assessment. Third, there would be value in including a matched comparison group of youth not receiving APMs. Ideally, the comparator group would comprise sociodemographically-matched youth with mental health disorders that do not require treatment with APMs, such as depression and/or anxiety. Though it is worthy to note that the reported energy intake was substantially higher for both males and females in this study compared to data from the general population aged 19–30 years (36); 13,651 vs. 11,004 kJ/day for males, and 11,513 vs. 7,863 kJ/day for women. This reinforces the effects that impairments in executive function/reward system and increased appetite have on energy intake in people with psychosis. Fourth, important covariables such as symptom level/functioning, economic status, education, marital status, and metabolic/cardiovascular comorbidities, were not able to be included in this analysis and should be considered for future studies. Fifth, we could not determine if the nutritional issues predated APM use. Dietary data in pre-medicated FEP are required, but there are significant pragmatic obstacles to collecting such data pre-treatment, due to the nature of untreated severe mental illness and compliance with nutritional assessment. Sixth, dietary intake was estimated using the diet history method, a subjective measure that could lead to selective underreporting. The gold-standard objective biomarkers and the doubly labeled water technique are expensive and intensive for both the researcher and participant, and therefore not feasible in all studies. Studies that include these objective measures would help confirm findings using subjective measures. The final limitation is the conservative model used to calculate discretionary food consumption; the benchmark was set at the upper limit recommended, which would generally be reserved for those with higher energy requirements. This may have underestimated discretionary food intake. The dietary methodology did not enable us to estimate intake of trans and saturated fats, and free sugars, particularly relevant given the high intakes of discretionary foods.

Comprehensive care of youth with severe mental illness should incorporate lifestyle interventions to ameliorate cardiometabolic risk factors. Weight-gain prevention interventions implemented after APM initiation have larger effect sizes, compared to weight-loss interventions in people with enduring severe mental illness (37). Recognition of rapid weight-gain and cardiometabolic health decline soon after APM-initiation, together with evidence that lifestyle interventions in early psychosis programs prevent such outcomes (22), underpin the Healthy Active Lives (HeAL) Declaration (38), that defines 5-year targets for key lifestyle factors contributing to poor cardiometabolic health in severe mental illness (www.iphys.org.au). These include specific dietitian-led interventions for weight management, which have been shown to be effective in severe mental illness (37). Further exploration is also needed for the use of adjunctive nutrients in people experiencing FEP as a potential method of improving symptoms and functioning (39).

While not explored in this study, it is important to note that people at ultra-high risk for psychosis and APM naïve people with FEP also have high rates of cardiometabolic abnormalities (40). This may be explained, at least in part, by the impaired executive functioning and reward system complicating the eating process and increasing the preference for non-nutritious foods high in sugar, salt and fat (14). Further exploration of the effectiveness of weight-gain preventative measures in those at ultra-high risk for psychosis and APM naïve people with FEP are needed.

In summary, this cross-sectional study suggests excessive consumption of discretionary and processed foods and numerous shortfalls in essential quality requirements are likely contributing not only to rapid weight gain, but also to other longer-term health sequelae in people in the early stages of APM treatment. Addressing the energy intake excesses observed in people with FEP receiving APM may assist in preventing weight-gain in the early stages of APM treatment. Larger, prospective studies, measuring dietary intake at multiple time points would assist in providing a greater understanding of the dietary intake, and its effects on health, in people with FEP.

## Author Contributions

ST, PW, AW, JC, and KS conceived the study and sought ethical approval. ST, RJ, TW, and ER completed the assessments. JC and JL provided psychiatry input and confirmed diagnoses. ST, PW, and KS analyzed the data. ST led manuscript preparation. PW, JC, JL, and KS added scientific intellect. RJ, TW, ER, and AW assisted with clinically relevant components. All authors approved the final version of the manuscript.

### Conflict of Interest Statement

The authors declare that the research was conducted in the absence of any commercial or financial relationships that could be construed as a potential conflict of interest.

## References

[1] Correll CU Robinson DG Schooler NR Brunette MF Mueser KT Rosenheck RA. Cardiometabolic risk in patients with first-episode schizophrenia spectrum disorders: baseline results from the RAISE-ETP study. JAMA Psychiatry (2014) 71:1350–63. 10.1001/jamapsychiatry.2014.1314 25321337

[2] Curtis J Henry C Watkins A Newall H Samaras K Ward PB. Metabolic abnormalities in an early psychosis service: a retrospective, naturalistic cross-sectional study. Earl Interv Psychiatry (2011) 5:108–14. 10.1111/j.1751-7893.2011.00262.x 21470374

[3] Zhang Q Deng C Huang X-F. The role of ghrelin signalling in second-generation antipsychotic-induced weight gain. Psychoneuroendocrinology (2013) 38:2423–38. 10.1016/j.psyneuen.2013.07.010 23953928

[4] Álvarez-Jiménez M González-Blanch C Crespo-Facorro B Hetrick S Rodriguez-Sánchez JM Pérez-Iglesias R. Antipsychotic-induced weight gain in chronic and first-episode psychotic disorders. CNS Drugs (2008) 22:547–62. 10.2165/00023210-200822070-00002 18547125

[5] Vancampfort D Wampers M Mitchell AJ Correll CU Herdt A Probst M. A meta-analysis of cardio-metabolic abnormalities in drug naïve, first-episode and multi-episode patients with schizophrenia versus general population controls. World Psychiatry (2013) 12:240–50. 10.1002/wps.20069 24096790 PMC3799255

[6] Brown S Kim M Mitchell C Inskip H. Twenty-five year mortality of a community cohort with schizophrenia. Br J Psychiatry (2010) 196:116–21. 10.1192/bjp.bp.109.067512 20118455 PMC4560167

[7] Newcomer JW. Antipsychotic medications: metabolic and cardiovascular risk. J Clin Psychiatry (2007) 68:8–13. 17539694

[8] Strassnig M Kotov R Cornaccio D Fochtmann L Harvey PD Bromet EJ 20-year progression of BMI in a county-wide cohort of people with schizophrenia and bipolar disorder identified at their first episode of psychosis. Bipolar Disord. (2017) 19:336–43. 10.1111/bdi.12505 28574189 PMC5568920

[9] Fountaine RJ Taylor AE Mancuso JP Greenway FL Byerley LO Smith SR. Increased food intake and energy expenditure following administration of olanzapine to healthy men. Obesity (2010) 18:1646–51. 10.1038/oby.2010.6 20134408

[10] Dipasquale S Pariante CM Dazzan P Aguglia E McGuire P Mondelli V. The dietary pattern of patients with schizophrenia: a systematic review. J Psychiatr Res. (2013) 47:197–207. 10.1016/j.jpsychires.2012.10.005 23153955

[11] Kluge M Schuld A Himmerich H Dalal M Schacht A Wehmeier PM. Clozapine and olanzapine are associated with food craving and binge eating: Results from a randomized double-blind study. J Clin Psychopharmacol. (2007) 27:662–6. 10.1097/jcp.0b013e31815a8872 18004133

[12] Stubbs B Williams J Gaughran F Craig T. How sedentary are people with psychosis? a systematic review and meta-analysis. Schizophr Res. (2016) 171:103–9. 10.1016/j.schres.2016.01.034 26805414

[13] Lett TAP Wallace TJM Chowdhury NI Tiwari AK Kennedy JL Müller DJ. Pharmacogenetics of antipsychotic-induced weight gain: review and clinical implications. Mol Psychiatry (2012) 17:242–66. 10.1038/mp.2011.109 21894153

[14] Minichino A Francesconi M Salatino A Delle Chiaie R Cadenhead K. Investigating the link between drug-naive first episode psychoses (FEPs), weight gain abnormalities and brain structural damages: relevance and implications for therapy. Prog Neuropsychopharmacol Biol Psychiatry (2017) 77:9–22. 10.1016/j.pnpbp.2017.03.020 28363765

[15] Knolle-Veentjer S Huth V Ferstl R Aldenhoff JB Hinze-Selch D. Delay of gratification and executive performance in individuals with schizophrenia: Putative role for eating behavior and body weight regulation. J Psychiatric Res. (2008) 42:98–105. 10.1016/j.jpsychires.2006.10.003 17126365

[16] Bauer UE Briss PA Goodman RA Bowman BA. Prevention of chronic disease in the 21st century: elimination of the leading preventable causes of premature death and disability in the USA. Lancet (2014) 384:45–52. 10.1016/S0140-6736(14)60648-6 24996589

[17] American Psychiatric Association Diagnostic and Statistical Manual of Mental Disorders (DSM-5®). Washington DC: American Psychiatric Pub (2013).

[18] Lachat C Hawwash D Ocké MC Berg C Forsum E Hörnell A Strengthening the reporting of observational studies in epidemiology–nutritional epidemiology (STROBE-nut): An extension of the STROBE statement. Nutr Bull. (2016) 41:240–51. 10.1371/journal.pmed.1002036 27587981 PMC4988500

[19] Burrows TL Martin RJ Collins CE. A systematic review of the validity of dietary assessment methods in children when compared with the method of doubly labeled water. J Am Diet Assoc. (2010) 110:1501–10. 10.1016/j.jada.2010.07.008 20869489

[20] Thompson FE Subar AF Dietary assessment methodology. In: Coulston ACB, editors. Nutrition in the Prevention and Treatment of Disease. 2nd edn. London: Elsevier (2008).

[21] Curtis J Newall HD Samaras K. The heart of the matter: cardiometabolic care in youth with psychosis. Earl Interv Psychiatry (2012) 6:347–53. 10.1111/j.1751-7893.2011.00315.x 22221395

[22] Curtis J Watkins A Rosenbaum S Teasdale S Kalucy M Samaras K. Evaluating an individualized lifestyle and life skills intervention to prevent antipsychotic-induced weight gain in first-episode psychosis. Earl Interv Psychiatry (2016) 10:267–76. 10.1111/eip.12230 25721464

[23] National Health Medical Research Council Australian Dietary Guidelines. Canberra: NHMRC (2013). Available online from: http://www.eatforhealth.gov.au.

[24] Schofield WN. Predicting basal metabolic rate, new standards and review of previous work. Hum Nutr Clin Nutr. (1984) 39:5–41. 4044297

[25] Black AE. Critical evaluation of energy intake using the Goldberg cut-off for energy intake: basal metabolic rate. A practical guide to its calculation, use and limitations. Int J Obesity (2000) 24:1119. 10.1038/sj.ijo.0801376 11033980

[26] Krenitsky J. Adjusted body weight, pro: evidence to support the use of adjusted body weight in calculating calorie requirements. Nutr Clin Pract. (2005) 20:468–73. 10.1177/0115426505020004468 16207686

[27] Goldberg G Black A Jebb S Cole T Murgatroyd P Coward W. Critical evaluation of energy intake data using fundamental principles of energy physiology: 1. Derivation of cut-off limits to identify under-recording. Eur J Clin Nutr (1991) 45:569–81. 1810719

[28] World Health Organisation Global Database on Body Mass Index. Geneva: WHO (2018) Available online at: https://www.who.int/gho/ncd/risk_factors/bmi_text/en/

[29] Alberti KGMM Zimmet P Shaw J. Metabolic syndrome - a new world-wide definition. A consensus statement from the international diabetes federation. Diabet Med. (2006) 23:469–80. 10.1111/j.1464-5491.2006.01858.x 16681555

[30] Hall KD Sacks G Chandramohan D Chow CC Wang YC Gortmaker SL. Quantification of the effect of energy imbalance on bodyweight. Lancet (2011) 378:826–37. 10.1016/S0140-6736(11)60812-X 21872751 PMC3880593

[31] Rosenbaum S Tiedemann A Sherrington C Curtis J Ward PB. Physical activity interventions for people with mental illness: a systematic review and meta-analysis. J Clin Psychiatry (2014) 75:964–74. 10.4088/JCP.13r08765 24813261

[32] Firth J Carney R Elliott R French P Parker S McIntyre R. Exercise as an intervention for first-episode psychosis: a feasibility study. Earl Interv Psychiatry (2016) 12:307–15. 10.1111/eip.12329 26987871 PMC6001796

[33] McArdle WD Katch FI Katch VL Energy expenditure at rest and during physical activity. In: McArdle WD Katch FI Katch VL, editors. Essentials of Exercise Physiology. 5th edn. Philidelphia, PA: Wolters Kluwer (2016).

[34] Rummel-Kluge C Komossa K Schwarz S Hunger H Schmid F Lobos CA. Head-to-head comparisons of metabolic side effects of second generation antipsychotics in the treatment of schizophrenia: a systematic review and meta-analysis. Schizophr Res. (2010) 123:225–33. 10.1016/j.schres.2010.07.012 20692814 PMC2957510

[35] Maayan L Correll CU. Weight gain and metabolic risks associated with antipsychotic medications in children and adolescents. J Child Adolesc Psychopharmacol. (2011) 21:517–35. 10.1089/cap.2011.0015 22166172

[36] Australian Bureua of Statistics (ABS) Australian Health Survey: Nutrition First Results - Foods Nutrients 2011-12. Canberra: ABS (2014). Available online at: http://www.abs.gov.au/ausstats/abs@.nsf/lookup/4364.0.55.007main+features12011-12

[37] Teasdale SB Ward PB Rosenbaum S Samaras K Stubbs B. Solving a weighty problem: systematic review and meta-analysis of nutrition interventions in severe mental illness. Br J Psychiatry (2017) 210:110–8. 10.1192/bjp.bp.115.177139 27810893

[38] Shiers D Curtis J. Cardiometabolic health in young people with psychosis. Lancet Psychiatry (2014) 1:492–4. 10.1016/S2215-0366(14)00072-8 26361295

[39] Firth J Rosenbaum S Ward PB Curtis J Teasdale SB Yung AR. Adjunctive nutrients in first episode psychosis: a systematic review of efficacy, tolerability and neurobiological mechanisms. Early Interv Psychiatry (2018) 12:774–83. 10.1111/eip.12544 29561067 PMC6175456

[40] Cadenhead KS Minichino A Kelsven S Addington J Bearden C Cannon TD. Metabolic abnormalities and low dietary Omega 3 are associated with symptom severity and worse functioning prior to the onset of psychosis:i findings from the North American Prodrome Longitudinal Studies Consortium. Schizophr Res. (2018) 10.1016/j.schres.2018.09.022. [Epub ahead of print]. 30249470 PMC6402991

