# Supplementary material for: Is Obesity in Young People With Psychosis a Foregone Conclusion? Markedly Excessive Energy Intake Is Evident Soon After Antipsychotic Initiation
Source: Front Psychiatry. 2018 Dec 24;9:725. doi: 10.3389/fpsyt.2018.00725 (PMC6312040; doi:10.3389/fpsyt.2018.00725)
Supplement: Supplementary file 1 [file Table_1.DOCX]

**SUPPLEMENTARY MATERIAL 1**

Schofield Equations:

| *Age* | *Male* | *Female* |
| --- | --- | --- |
| 10-18 | ((0.074 x W) + 2.754) x PAL | ((0.056 x W) + 2.898) x PAL |
| 18-30 | ((0.063 x W) + 2.896) x PAL | ((0.062 x W) + 2.036) x PAL |
| 30-60 | ((0.048 x W) + 3.653) x PAL | ((0.034 x W) + 3.538) x PAL |
| >60 | ((0.049 x W) + 2.459) x PAL | ((0.038 x W) + 2.755) x PAL |

W = weight (kg), PAL = physical activity level

* no injury factor was included in these analyses

Example of estimated energy requirement:

Miss A

25yo

Height = 172cm

Weight = 94.1kg

Adjusted ideal body weight (AIBW) = 74kg

Physical Activity Level = sedentary

Schofield equation for 25yo female;

(0.062 x Weight or AIBW) + 2.036 x PAL

((0.062 x 74) + 2.036) x 1.4 = **9,274kJ/day**
